# Effect of budesonide/glycopyrrolate/formoterol fumarate metered dose inhaler combined with nasal high-flow nasal cannula on elderly patients with COPD and respiratory failure

**DOI:** 10.12669/pjms.40.3.8395

**Published:** 2024

**Authors:** Feiyan Hu, Feijing Lv

**Affiliations:** 1 Feiyan Hu, Department of Respiratory and Critical Care Medicine. Yongkang First People’s Hospital, Yongkang, Zhejiang Province 321300, P.R. China; 2 Feijing Lv, Department of Emergency General Ward, Yongkang First People’s Hospital, Yongkang, Zhejiang Province 321300, P.R. China

**Keywords:** Budesonide/glycopyrrolate/formoterol fumarate metered dose inhaler, High-flow nasal cannula, Chronic obstructive pulmonary disease, Respiratory failure

## Abstract

**Objective:**

To explore the clinical effect of budesonide/glycopyrrolate/formoterol fumarate metered dose inhaler (BGF MDI) combined with high-flow nasal cannula (HFNC) in the treatment of elderly patients with chronic obstructive pulmonary disease (COPD) and respiratory failure.

**Methods:**

The clinical records of 94 elderly patients with COPD and respiratory failure who were treated in Yongkang First People’s Hospital from February 2022 to January 2023 were retrospectively selected. Among them, 46 patients received HFNC alone (Control-group) and 48 patients received HFNC combined with BGF MDI (Study-group). The treatment effect, arterial blood gas status, pulmonary function, and acute physiology and chronic health evaluation (APACHE) II score before and after treatment were analyzed in both groups.

**Results:**

The total efficacy of treatment in the Study-group (95.8%) was higher than that in the Control-group (78.3%) (P<0.05). After treatment, the partial pressure of arterial carbon dioxide (PaCO_2_), residual volume, and APACHE II scores in the two groups decreased compared to those before treatment, with the Study-group lower overall. However, arterial oxygen saturation (SaO_2_), oxygen partial pressure (PaO_2_), the percentage of peak expiratory flow (PEF), and forced expiratory volume in one second (FEV_1_) as percent of predicted (%FEV_1_) were higher than before treatment, and higher in the Study-group (P<0.05).

**Conclusions:**

Compared with HFNC alone, BGF MDI combined with HFNC can effectively regulate the arterial blood gas status of elderly patients with COPD and respiratory failure, restore pulmonary function, and improve the overall treatment effect.

## INTRODUCTION

Chronic obstructive pulmonary disease (COPD) is a respiratory disease prevalent in the elderly population.^1^ Patients with COPD have limited airflow, which can easily recur without timely and effective intervention, leading to respiratory failure, shortness of breath, and dyspnea. In severe cases, patients with advanced COPD and acute or chronic respiratory failure can be deadly.^1^,^2^ Therefore, it is important to take early effective measures to intervene in patients with COPD and respiratory failure.^1^-^3^

Current treatment for COPD with respiratory failure involves the use of a non-invasive ventilator, which can quickly and effectively improve ventilation status and alleviate breathing difficulties.^4^ However, this method can easily cause aspiration, bloating, dry mouth and nose, or mask a pressure injury.^2^,^4^ High-flow nasal cannula (HFNC) is also commonly used in the treatment of respiratory diseases.^5^ This treatment heats and humidifies inhaled gases, allowing patients to freely eat and breathe, which is more in line with their physiological needs, and can provide high concentration medication and oxygen concentration accuracy, which is conducive to improving ventilation function.^5^,^6^ Budesonide/glycopyrrolate/formoterol fumarate metered dose inhaler (BGF MDI) is a new therapeutic drug for respiratory diseases, which combines the advantages of formoterol, glucuronium bromide, and budesonide, and can synergistically improve COPD.^7^ Based on the therapeutic effects of HFNC and BGF MDI, we supposed that the combination of the two may further improve their clinical effects. Moreover, there is limited data on this combination treatment. Therefore, the aim of this study was to assess the clinical value of BGF MDI combined with HFNC in the treatment of elderly patients with COPD and respiratory failure, the specific records are reviewed and analyzed as follows.

## METHODS

The clinical records of 94 elderly patients with COPD and respiratory failure who were treated in Yongkang First People’s Hospital from February 2022 to January 2023 were retrospectively selected, including 55 males and 39 females. Forty-six patients received HFNC treatment were assigned to the Control-group, while 48 patients received HFNC combined with BGF MDI were assigned to the Study-group.

### Ethical Approval

This study was approved by the Medical Ethics Committee of Yongkang First People’s Hospital (No. 20230329, date: 2023-03-29).

### Inclusion criteria:

- Comply with the diagnostic criteria for COPD.^8^
- Meet the diagnostic criteria for respiratory failure.^9^
- Age ≥ 60 years old.
- Have complete clinical data.

### Exclusion criteria:

- Patients with abnormal kidney and liver function.
- Presence of benign and malignant tumors.
- Existence of infectious diseases.
- Presence of diseases of the blood and endocrine system.
- Presence of other respiratory diseases (such as pneumonia or bronchial asthma)
- Persistent difficulty in discharging airway secretions.
- Patients who are allergic to or have contraindications to BGF MDI
- Patients with mental illness.

### Control-group (HFNC alone)

An OH-70B nasal high flow humidifying oxygen therapy system (Shanghai Sibairui, China) was used and the oxygen flow rate was set to 20-40L/minutes. The oxygen concentration was set to 30%-50%, the gas temperature was maintained at 37°C, and the relative humidity of the gas was controlled at 100%. During treatment, the patients’ vital signs and blood gas status were closely monitored. If their condition worsened, symptomatic interventions such as endotracheal intubation and mechanical ventilation were given immediately.

### Study-group (HFNC combined with BGF MDI)

### On the basis of the control group, the study group also received BGF MDI

Treatment was given two times a day. Each bottle contained 120 ounces: 160μg of budesonide, 7.2μg of glycopyrrolate, and 4.8μg of formoterol fumarate. The manufacturer is AstraZeneca Dunkerque Production (Approval No.: H20190063).

### Patient characteristics and related indicators before and after treatment:

### Treatment effect

The treatment effect was divided into three levels: 1) significant effect: the arterial blood gas, return to normal functional status, and the disappearance of clinical symptoms; 2) effective: an improvement in arterial blood gas and a functional status ≥ 50% along with a significant improvement of clinical symptoms; 3) ineffective: if the improvement in arterial blood gas and functional status was less than 50%, and the clinical symptoms were not relieved. Total effective rate = (significant + effective) / total number of cases × 100%.

### Arterial blood gas status

Arterial blood was collected and the levels of PaCO_2_, SaO_2_, and PaO_2_ were measured using ABL-90 (Radiometer, Denmark) blood gas analyzer.

### Pulmonary function

The levels of PEF, %FEV_1_, and RV, were measured using the German Yeger pulmonary function instrument.

### Acute Physiology and Chronic Health Evaluation (APACHE) II score

APACHE II score system was used to assess disease severity. It ranges from 0 to 71, with higher scores associated with a higher risk of hospital death.

### Statistical Analysis

SPSS 25.0 (SPSS Inc., Chicago, IL, USA) was used for data analysis. Continuous variables were described as mean ± standard deviation [SD]. Discrete variables were described as counts. Chi-square tests were conducted to compare discrete variables and student’s *t*-tests were used to compare continuous variables. The clinical efficacy was compared using Chi-square tests, while the level of arterial blood gas status and pulmonary function were compared using student’s *t*-tests. All statistical tests were 2-sided, and statistical significance was set at *P*<0.05.

## RESULTS

A total of 94 patients were included in this study. The average patient age was 71.16 ± 5.64 years. The average length of COPD was 4.97 ± 1.87 years, while the average APACHE II score was 21.21 ± 2.40 points. There were 46 cases in the Control-group, including 25 males and 21 females. The average age was 71.80±5.46 years, the average length of COPD was 4.70±1.98 years, and the average APACHE II score was 21.65±2.30 points. The Study-group consisted of 48 patients, including 30 males and 18 females. The average age was 70.54±5.79 years, the average length of COPD was 5.22±1.74 years, and the average APACHE II score was 20.79±2.44 points. There was no significant difference between the two groups in baseline characteristics such as gender, age, length of COPD, and APACHE II score (*P*>0.05) (Table-I). The total efficacy of treatment in the Study-group (95.8%) was higher than that in the Control-group (78.3%) (P<0.05; Table-II).

**Table-I:** Baseline characteristics of the Study group vs the Control group

Group	n	Gender (male/female)	Age (year)	Length of COPD (year)	APACHE II score
Study-group	48	30/18	70.54±5.79	5.22±1.74	20.79±2.44
Control-group	46	25/21	71.80±5.46	4.70±1.98	21.65±2.30
*χ^2^*/*t*		0.643	-1.086	1.390	-1.757
*P*		0.423	0.696	0.232	0.464

**Table-II:** Clinical efficacy of the Study group vs the Control group.

Group	n	Significant effect	Effective	Invalid	Total effective rate
Study-group	48	28(58.3)	18(37.5)	2(4.2)	45(95.8)
Control-group	46	19(41.3)	17(37.0)	10(21.7)	36(78.3)
*χ^2^*					7.046
*P*					0.030

Before treatment, there was no difference in PaCO_2_, SaO_2_, or PaO_2_ between the two groups (P>0.05). After treatment, PaCO_2_ decreased in both groups compared to before treatment, and was lower in the Study-group. However, the SaO_2_ and PaO_2_ were higher than before treatment, and higher in the Study-group (P<0.05; Table-III).

**Table-III:** Arterial blood gas status of the Study group vs the Control group.

Time	Group	n	PaCO_2_ (mmHg)	SaO_2_ (%)	PaO_2_ (mmHg)
Before treatment	Study-group	48	75.54±6.33	79.25±4.97	40.25±3.33
	Control-group	46	76.87±6.10	80.30±5.89	39.37±3.16
	t		-1.035	-0.939	1.312
	P		0.303	0.350	0.193
After treatment	Study-group	48	48.02±5.49^a^	95.21±5.81^a^	85.64±5.93^a^
	Control-group	46	56.87±5.26^a^	89.24±6.60^a^	78.72±5.26^a^
	t		-7.969	4.661	5.979
	P		<0.001	<0.001	<0.001

***Note:*** Compared with this group before treatment,

a P<0.05.

There was no difference in PEF, % FEV_1_, and RV between the two groups before treatment (P>0.05). After treatment, PEF and % FEV_1_ were higher in the two groups than before treatment, and higher in the Study-group. RV was lower than before treatment and lower in the Study-group (P<0.05; Table-IV). Similarly, before treatment, there was no difference in the APACHE II scores between the two groups (P>0.05). After treatment, the APACHE II scores in the two groups decreased compared to before treatment, and the Study-group was lower (P<0.05; Table-V).

**Table-IV:** Lung function of the Study group vs the Control group.

Time	Group	n	PEF (L)	%FEV1	RV (L/s)
Before treatment	Study-group	48	3.25±0.51	61.23±6.10	3.31±0.86
	Control-group	46	3.35±0.55	59.78±6.69	3.20±10.85
	t		-0.897	1.096	0.601
	P		0.372	0.276	0.549
After treatment	Study-group	48	4.37±0.64^a^	76.12±8.03^a^	2.40±0.52^a^
	Control-group	46	3.95±0.67^a^	70.24±7.44^a^	2.99±0.64^a^
	t		3.101	3.684	-4.922
	P		0.003	0.001	<0.001

***Note:*** Compared with this group before treatment,

a P<0.05.

**Table-V:** APACHE II scores of the Study group vs the Control group.

Group	n	Before treatment	After treatment	t	P
Study-group	48	20.79±2.44	11.96±1.96	51.385	<0.001
Control-group	46	21.65±2.30	13.93±2.69	18.421	<0.001
*t*		-1.757	-4.082		
*P*		0.082	<0.001		

## DISCUSSION

The results of this study showed that compared to HFNC alone, BGF MDI combined with HFNC is more effective in treating elderly patients with COPD and respiratory failure. Furthermore, this treatment combination can more effectively improve lung function and arterial blood gas status compared to HFNC alone.

Previous work by Tan et al.^10^ showed that in patients with COPD, severe hypercapnia and respiratory failure who received invasive ventilation, compared to non-invasive ventilation, HFNC did not increase the risk of treatment failure, and was also better tolerated by patients. Sun et al.^11^ used noninvasive ventilation and HFNC to treat patients with COPD and acute respiratory failure. They found that the success rate of HFNC treatment was similar to that of non-invasive ventilation, and patients who adopted HFNC showed lower nursing intervention and skin injury event incidence rates. Chohnabayashi et al.^12^ screened 104 COPD patients with daytime hypercapnia and administered long-term oxygen therapy and HFNC. The results confirmed that HFNC can effectively reduce the risk of moderate to severe deterioration of the COPD, and has more significant advantages in improving patients’ health-related quality of life, blood oxygen saturation, and lung function. Research by Xu et al.^13^ showed that compared to non-invasive ventilation, HFNC can shorten the hospitalization time of patients with COPD and respiratory failure, reduce PaCO_2_, and help reduce the risk of skin damage at the nasal surface. There was no significant difference between the two groups in mortality and the incidence of tracheal intubation. Cortegiani et al.^14^ used non-invasive ventilation and HFNC to treat patients with COPD and acute hypercapnia exacerbation, and the results confirmed that the effects of both methods were equivalent. Research by Miquel Ferrer et al.^15^ has also confirmed that HFNC has high application value in patients with COPD, can improve gas exchange, alleviate breathing difficulties, and has important significance in reducing disease mortality and tracheal intubation rates. Our findings are consistent with the above studies.

Research suggests that the application of HFNC in COPD with respiratory failure is valuable, but in clinical practice, this has yet to be fully established.^16^,^17^ Work by Gao et al.^16^ indicates that comprehensive intervention with inhaled drugs based on non-invasive ventilation can further improve symptoms such as dyspnea, expectoration, and cough, regulate blood gas status, and promote pulmonary function recovery. BGF MDI is a long-acting, inhaled aerosol, of which formoterol fumarate is a β2 receptor long-acting agonist, glycopyrrolate is a new type of acetylcholinergic receptor blocker with high drug resistance and long effect, and budesonide is a glucocorticoid with anti-inflammatory activity.^18^ The long-term combined delivery of drugs through co-suspension drug delivery technology can exert the effects of relaxing bronchial smooth muscle, regulating spasm, and expanding the airway by inhibiting the activation of bronchial smooth muscle β2 receptor, bronchial smooth muscle M3 acetylcholine receptor, and providing anti-inflammatory effects.^17^,^19^ At the same time, there are certain differences in the density of β2 receptors and M3 acetylcholine receptors in the peripheral and central airways of the lung. Therefore, taking budesonide can also relax the smooth muscle of the peripheral and central airways, which is conducive to further regulating lung function.^19^,^20^ The combined BGF MDI can enhance the ability to jointly resist lung injury, and improve smooth muscle reactivity, and respiratory muscle strength. Research by Heo YA et al.^18^ also confirmed that among BGF MDI components, formoterol fumarate can relax smooth muscle and effectively alleviate bronchospasm. Budesonide can prevent airway remodeling, thereby exerting an anti-asthmatic effect, while glycopyrrolate has a good bronchodilator effect. The combined action of the three drugs can more effectively alleviate symptoms such as dyspnea in patients, inhibit the process of lung tissue remodeling, and thereby improve lung function in patients.

### Limitations

This study retrospectively examined 94 patients with COPD from our hospital, a sample size which could be improved further. Additionally, the indicators included in this study was limited and no follow-up data were analyzed.

## CONCLUSION

Compared with HFNC alone, BGF MDI combined with HFNC in the treatment of elderly patient with COPD and respiratory failure can more effectively regulate arterial blood gas status, restore lung function, improve health, and enhance the overall treatment effect.

### Authors’ contributions:

**FH:** Conceived and designed the study.

**FH** and **FL:** Collected the data and performed the analysis.

**FH:** Was involved in the writing of the manuscript and is responsible for the integrity of the study.

All authors have read and approved the final manuscript.
